# For better or worse: Relationship change in Thailand during COVID-19

**DOI:** 10.1371/journal.pone.0264614

**Published:** 2022-05-25

**Authors:** Juthatip Wiwattanapantuwong, Arunya Tuicomepee, Panrapee Suttiwan, Rewadee Watakakosol, Menachem Ben-Ezra, Robin Goodwin

**Affiliations:** 1 Faculty of Psychology, Chulalongkorn University, Bangkok, Thailand; 2 Department of Psychology, Graduate School of Arts and Letters, Tohoku University, Sendai, Miyagi, Japan; 3 LIFE Di Centre, Chulalongkorn University, Bangkok, Thailand; 4 School of Social Work, Faculty of Social Science and Humanities, Ariel University, Ariel, Israel; 5 Department of Psychology, Faculty of Science, Engineering and Medicine, University of Warwick, Coventry, United Kingdom; Konkuk University, REPUBLIC OF KOREA

## Abstract

**Objective:**

Novel infectious diseases have the potential to both strengthen or weaken interpersonal relationships within a society. In a collectivist setting such as Thailand amplification of relationships may be particularly marked, but may be associated with individual factors, including personal values and perceived control over the virus.

**Methods:**

A national on-street survey in Thailand (April 2020, N = 1,000), collected data from five regions across the country (response rate 82.6%). Participants reported demographics, anxiety, perceived control, and personal values of security and universalism, and indicated changes, from negative to positive, across four relationship types (relationship partners, family, friendships and neighbourhood).

**Results:**

While relationship changes were small overall, there was an improvement in close relations (partners, family members) but not amongst friends and neighbours. Respondents who were married without children recorded less enhancement of partnerships, friendships and neighbourhood relations. Those with less perceived control over the infection reported relationship decline, while single people reported fewer positive changes in their partnership or family relations. Multiple regression analyses demonstrated the prioritisation of security was associated with a decline in each of the relationships, while universalism was positively associated with change in the family, in friendships and neighbourly relations.

**Conclusions:**

Personal values and marital status may impact on relationship functioning during a national health crisis. These issues should be considered by clinicians and health practitioners when trying to assist those struggling with interpersonal relations during a pandemic.

## Introduction

Newly emergent infectious diseases, such as COVID-19, pose serious health and economic challenges. However, the impact of such a threat on the quality of interpersonal relationships is less clear, with lockdowns creating new and altered proximities as well as distances, and relationship changes partly dependent on context (e.g., income, minority status) and individual vulnerabilities (e.g., depression, attachment insecurity) [1]. On the one hand, the anxiety associated with a potentially life-threatening virus can seriously disrupt our regular interactions with others. Restrictions on movement has been linked to depression and PTSD [2] while the closures of schools and educational institutions interrupts individuals’ ability to work or study. Disruption to normal domestic practices can lead to marital discord and increased domestic abuse [3–5]. Community relations can also become strained as existing tensions are exacerbated [6–8]. Even where reliable online communications are available, quality of this interaction is likely to be reduced when compared with face-to-face conversation [9]. At the same time, a sense of shared national threat can promote common solidarities [10]. Romantic relationships can benefit as couples seek security during a time of existential concern [11]. Families may become of greater significance, particularly during confinement [12, 13]. Shared trauma can stimulate new liaisons and support networks within and across communities [14, 15]. Survey evidence from the UK suggest more than 70% of people experienced an increase in kindness and a greater sense of national unity as a result of the new coronavirus [4].

In this paper we examine perceived relationship change since the onset of COVID-19, and its associations with anxiety. We draw upon the Social Convoy Model [16], as a heuristic framework for conceptualizing social relationships. This model maps relationships into a circle of close relationships (relationship partners and family), a second more distal circle (e.g., friends), and a third, less intimate circle (e.g., co-workers) [17]. We anticipate that, overall, relationships between couples will decline during the pandemic [8], although this decline may be buffered by the presence of children [8]. Similarly, relations with family members may also be expected to be strained during a pandemic and might therefore expect to be poorer as a result [18, 19]. Naser and colleagues further found that social relations of friends and colleagues were negatively affected by the pandemic, with 37.8% of participants reporting that these social relationships became weaker [20].

Research during the pandemic has also shown that experience following the pandemic may be additionally fruitfully examined as an interaction between personality and societal circumstances [12]. Those particularly anxious about the virus may seek refuge, particularly in their partners—hence close relationship may buffer against this for the most anxious [11]. Previous research found that respondents who were confined during COVID lockdown with an inner circle group (e.g., family, roommates, or friends) reported positive relationship changes [13, 21]. Similarly, just as with anxiety, those who perceive they have least control over infection will seek reassurance from their close relationships [11]. However, individual vulnerability, such as high levels of anxiety, may actually worsen more distal relationships [1]. Work on previous infectious diseases suggests a negative association between anxiety and interactions with others with whom the individual is not personally familiar [22]. We also consider the role played by individual values. Schwartz offers a circumplex model of personal values which distinguishes between social and person-focused values [23, 24]. We consider two key values; universalism and security value, core values which are closely linked to relations between the self and others. Those who value *universalism* prioritise social justice, broad-mindedness, wisdom, and the protection of the welfare of all; individuals prioritising *security* emphasise safety and stability in the society, including the safety of the self, family, and nation [25]. While both values have been found to increase during COVID-19 [26, 27], these values may have very different consequences for personal relationships. As universalism recognises the vulnerabilities of all we anticipate universalism will be positively associated with relationship enhancement with both close others and the wider community during a time of crisis. A fear of insecurity and contagion, however, may inhibit those high on security in their communication with both close others and others in their communities.

We test these associations in a country which, thus far, has received comparatively little attention when considering psychological response to COVID-19. As home to the world’s most frequently visited city (Bangkok) Thailand was always vulnerable to the emergence of a new highly infectious pathogen. As the first country to identify the SARS-CoV-2 infection outside of China (January 8th, 2020) [28], and a nation heavily dependent on the tourism industry, the country was rapidly threatened by significant economic decline [29, 30]. As elsewhere, the new coronavirus led to a state of emergency and an initially ‘soft’ lockdown, introduced nationally on 26th March 2020. While important everywhere, interpersonal relations are central to many aspects of Thai life. A highly collectivistic nation, individuals in Thailand are strongly integrated into extended family networks [31]. We consider relationship changes and its associated correlates using data from a national on-street survey recruited from across Thailand four months into the COVID-19 pandemic.

## Method

Research was conducted in accordance with the World Medical Association Declaration of Helsinki. Following ethical approval from Chulalongkorn University (COA No. 052/2020) data was collected in the two weeks between April 20- May 3, 2020. During that time confirmed cases of COVID-19 rose from 2792 to 2987, with deaths from the coronavirus increasing from 47 to 54.

An established Thai survey company (‘Blue Eagle Eye’) collected data from across Thailand (Table 1). Following a cognitive interview with six participants to trial the questions trained interviewers approached one in three pedestrians passing a randomly pre-determined point on regional shopping streets or near regional bus stations or local markets. Interviews took approximately six minutes. Interviewers used appropriate personal protective equipment (including facemasks and hand sanitizers) and maintained physical distance from interviewees at all times, in line with guidance from Thai national health authorities. At the time of study there were no restrictions on movements besides a late evening curfew. If interviewers or interviewees displayed any of an expanded list of symptoms recognised by the Thai government as indicative of potential COVID-19 (e.g. fever, cough, shortness of breath) the interview was immediately terminated.

**Table 1:** Participant characteristics, psychological distress, values and relationship change.

	Thailand (N = 1000)			
**Demographics**	Mean	SD	N	%
Age, Years	39.09	14.01		
Sex, Female			504	50.4
**Marital Status**				
Single			303	30.3
Married no children			98	9.8
Married with children			560	56.0
Divorced/widowed no children			11	1.1
Divorced/widowed with children			28	2.8
**Region of Thailand**				
Bangkok			400	40.0
Khonkaen (Northeast)			150	15.0
Chiangmai (North)			150	15.0
Chonburi (East)			150	15.0
Hat Yai (Southern)			150	15.0
**Occupation (where N> 10)**				
Blue collar			367	36.7
Student			136	13.6
Company worker			102	10.2
Retired			98	9.8
Unemployed			86	8.6
Freelance			75	7.5
Business owner			71	7.1
Housewife			33	3.3
Government officer			27	2.7
**Anxiety (low (1), moderate (2), high (3))**				
About personally catching COVID -19	2.06	.54		
Your family and closest friends about catching COVID -19	2.07	.59		
About infecting others	1.79	.64		
Mean score (all 3 above)	1.97	.50		
**Control**				
Likelihood of you getting infected by the virus? ((none (1), a little (2), great deal (3))	2.33	.58		
**Relationship change as a result of COVID-19 ((1) worse (2) same (3) better)**				
Partnerships (in relationship, N = 811), % worse, same, better	2.22	.43	3, 624, 184	.4, 77.3, 22.7
Family change	2.21	.43	8, 776, 216	.8, 77.6, 21.6
Friends change	1.97	.32	64, 898, 38	6.4, 89.8, 3.8
Neighbourhoods	1.96	.27	60, 925, 15	6.0, 92.5, 1.5
**Personal Values (1 (not at all) to 10 (very much so)**				
*Security*	16.99	1.90		
It is important for the government to make sure everyone is safe	8.41	1.12		
It is important to live in safe and secure surroundings	8.58	1.08		
*Universalism*	16.54	2.04		
It is important to make sure people are treated equally and have equal opportunities	8.39	1.25		
It is important to understand different people	8.15	1.29		

One thousand two hundred and eleven respondents were approached and explained the purpose of the study. One hundred and eight-nine refused to participate (15.6%), the remainder provided oral consent. We omitted 22 participants (1.8%) who did not complete the interview, using data from the 1,000 interviewees (82.6%) who answered all the questions. Data validation was by inspectors who randomly appeared and observed 30% of the total interviews.

Participants were 50.4% female; ages ranged from 16 to 77 (median age 38; national median for Thailand is 39 [32]). Responses were collected from five regions, chosen to represent the country as a whole (400 respondents from Bangkok, 150 from regions in the Northeast, North, East and South), with data collected from five of the thirteen largest cities in Thailand (Table 1).

Alongside demographics (age, sex, marital status, occupation and region) we used three items to assess anxiety, drawing on previous work on avian influenza (subtype AH7N9) [22, 33] and adapted to allow for the non-symptomatic spread of the disease. Items asked 1: *How anxious are you about catching COVID-19*; 2: *How anxious are you about your closest friends and family catching COVID-19*?; 3: *How anxious are you about accidentally infecting others with the virus*? *(when you pass it on and maybe don’t realise you are ill)*. Items were coded as (1) *not at all anxious*, (2) *moderately anxious* (3) *very anxious*. Given the strong positive correlation between items we combined the three into a single anxiety scale (α = .80). Perceived control over infection was assessed by a single item: *How much control do you think over the likelihood of you getting infected by the virus*? ((1) *no control* (2) *a little control* (3) *a great deal of control*)). Values were assessed on four, ten-item items (from 1 not at all to 10 very much so): security values by two items (1) *It is important for the government to make sure everyone is safe* (2) *It is important to live in safe and secure surroundings*; universalism by (1) *It is important to make sure people are treated equally and have equal opportunities* and (2) *It is important to understand different people*. Relationship change since the start of COVID-19 was measured by four items, asking *How has your relationship with your … intimate partner (family*, *friends*, *neighbours) changed*? ((1) *worse* (2) *same* (3) *better*). Change in partner relationships were measured only for those 811 participants in such a relationship. All questionnaire items were translated using a committee of four bi-lingual English-Thai speakers based at (blinded) University.

### Analysis

Zero-order correlations report descriptive associations between relationship change and anxiety, perceived control over infection, and the two values (security and universalism). Moderation effects of the two values on the associations between anxiety and relationship change were tested using SPSS Process (model 1), conducting this separately for the inner circle of relationships (i.e., partnerships and family members) and the outer circle of relations (friends and neighbour). Multivariate logistic regressions for each of the four relationships (partners, family relations, friendships, neighbours), simultaneously entered as predictors (1) age, (2) sex, (3) marital status (with married with children as reference) (4) anxiety, (5) security and (6) universalism.

## Result

**Fig 1:**
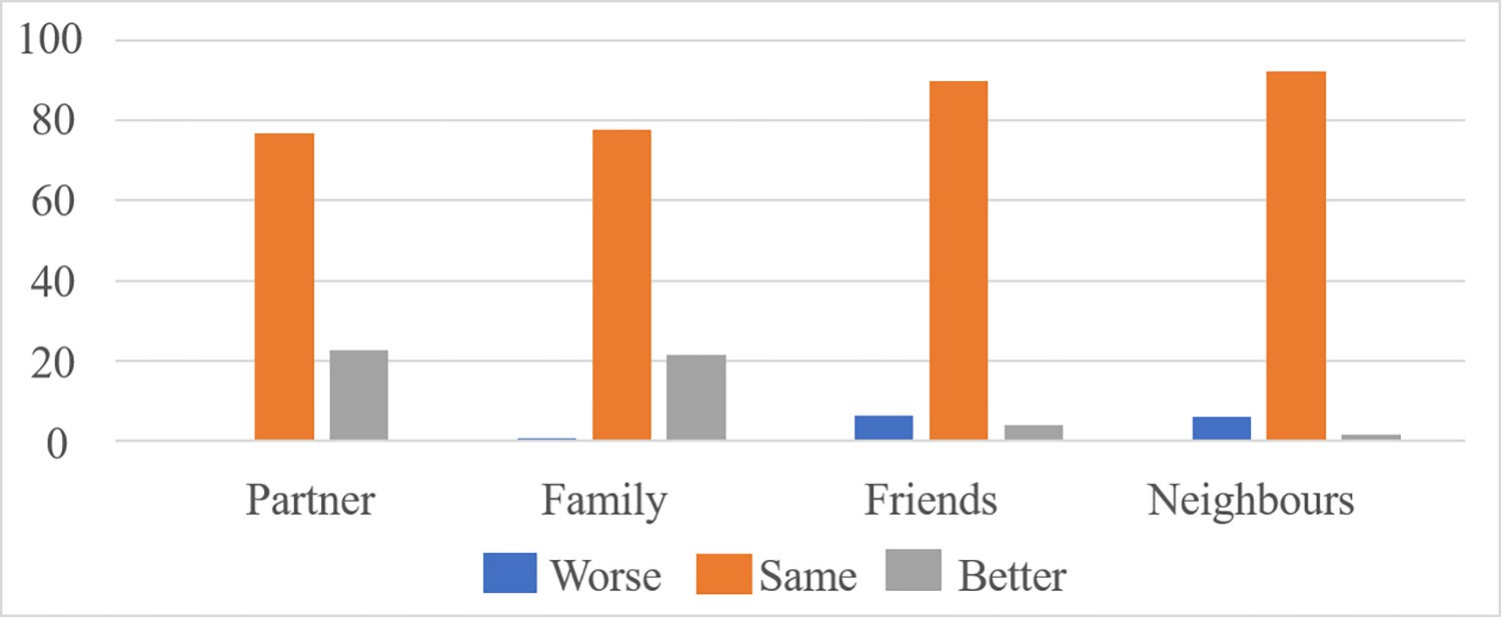
Percentage relationship change since COVID-19, by relationship type.

Averaging scores across all relationships, participants reported little change since the start of COVID-19 (M = 2.08, SD = 0.27, where 1 indicates decline, 2 no change, 3 an improvement). Fig 1 shows percentage change by relationship type. Respondents reported either enhancement (184, 22.7%) or no change (624, 77.3%) in their relations with their partners, with only 3 participants (.4%) reporting a decline. Similarly, 776 (77.6%) reported no change in their family relations, 216 (21.6%) an improvement in their relations, with 8 (,8%) indicating a decline. Most respondents reported no change in relations with friends or neighbours (898 (89.8%); 925 (92.5%), respectively) with only small percentages reporting an enhancement (38 (3.8%), 15 (1.5%)) or a decline ((64 (6.4%), 60 (6.0%)). To examine relationship changes by overall networks, we combined partners and family members into “an inner circle group” with friends and neighbours allocated to another, second/third circles group. Comparing changes in relationships between the two groupings indicated positive change was greatest amongst those in the “inner circle” (Ms = 2.22 vs. 1.96; t = 17.56, p = .001).

We then considered associations between relationship change and perception of the virus and respondent demographics and values. There were no significant zero-order correlations between averaged relationship change and anxiety (r (1000) = -.03, p = .41) or universalism (r (1000) = -.03, p = .38), but there was a small positive association between relationship change and perceived control (r (1000) = .06, p = .05) and a negative association between relationship change and security (r (1000) = -.16, p = .001). There was no significant correlation between anxiety and security (r (1000) = -.00, p = .95), but anxiety and universalism were negatively associated (r (1000) = -.08, p = .011).

Multiple regressions for each relationship included as covariates age, sex, marital status (dummy coded), anxiety, perceived control, and the two values (Table 2). Participants concerned about security were more likely to report negative change in each of their four relationships (partners (B = -.06, t = -5.89, p = .001, 95% CI (-.08, -.04), β = -.27), family relations (B = -.05, t = -5.22, p = .001, 95%CI (-.07, -.03), β = -.21), friendships (B = -.02, t = -3.30, p = .001, 95%CI (-.04, -.01), β = -.14), neighbourhood relations (B = -.02, t = -2.68, p = .01, 95%CI (-.03, -.004), β = -.11). Universalistic values were associated with enhanced relationships for all but partnerships (B = .01, t = 1.55, p = .12, 95% CI (.00, .03), β = .07; B = .02, t = 2.02, p = .04, 95%CI (.00, .03), β = .08; B = .02, t = 2.71, p = .01, 95%CI (.01, .03), β = .11; B = .01, t = 2.32, p = .02, 95%CI (.00, .02), β = .10, for partners, family relations, friends and neighbours respectively). Perceived control over the virus was positively associated with enhanced partner relationships (B = .11, t = 4.18, p = .001, 95%CI (.06, .17), β = .16). Compared to married respondents with children, single respondents reported less positive changes in their partnerships (B = -.15, t = -3.12, p = .002, 95%CI (-.238, -.054), β = -.13) and family relations (B = -.08, t = -2.19, p = .03, 95%CI (-.16, -.01), β = -.09); those married but without children less enhancement of their partnerships (B = -.09, t = -2.01, p = .04, 95%CI (-.19, -.002), β = -.07), friends (B = -.11, t = -3.14, p = .002, 95%CI (-.18, -.04), β = -.10) and neighbourhood interactions (B = -.09, t = -.2.81, p = .005, 95%CI (-.14, -.03), β = -.09). Results showed a positive association between anxiety and relationship change only for family relationships (B = .06, t = 1.97 p = .048, 95% CI (.00-.12), β = .07).

**Table 2:** Anxiety, values, demographics and relationship change.

	Relationships change among (B (95%CI))			
	Partners	Family	Friends	Neighbours
*Demographics*				
Age	-.00 (.00, .00)	-.00 (.00, .00)	-.00 (.00, .00)	-.00 (-.00, .00)
Sex (female)	.04 (-.02, .09)	.04 (-.01, .09)	.01 (-.03, .05)	.02 (-.01, .06)
Relationship Status (ref: married with children)				
Single	-.15** **(-.24, -.05)**	-.08* **(-.16, -.01)**	.05 (-.01, .10)	.02 (-.03, .07)
Married, no children	-.09* **(-.19, -.002)**	-.05 (-.14, .05)	**-.11** ** **(-.18, -.04)**	**-.09** ** **(-.14, -.03)**
Divorced/widowed no children	-.02 (-.35, .31)	-.05 (-.30, .20)	.08 (-.11, .27)	.08 (-.09, .24)
Divorced/Widowed with children	-.13 (-.40, .14)	-.12 (-.28, .05)	-.03 (-.15, .09)	.01 (-.10, .11)
*Psychological variables*				
Anxiety	.05 (-.02, .11)	**.06** * **(.00, .12)**	-.03 (-.08, .01)	-.02 (-.06, .02)
Control	.11*** **(.06,.17)**	.05 (.00, .10)	.00 (-.04, .03)	.01 (-.02, .04)
Security	-.06*** **(-.08, -.04)**	-.05*** **(-.07, -.03)**	-.02*** **(-.04, -.01)**	-.02** **(-.03, -.004)**
Universalism	.01 (.00, .03)	.02* **(.00, .03)**	.02** **(.01, .03)**	.01* **(.00, .02)**

*** p < .001

**p < .01

*p < .05. p < .05 or below in bold.

We ran regression analyses assessing the association of anxiety, two values (security and universalism), and change in relationship with people from the inner and outer circles separately. For intimate partner and family relationship change, security negatively predicted change (β = -.04, t = -6.20, p = .0001, 95%CI (-.05, -.03)) with no interaction between anxiety and security. This shows that the association between anxiety and relationship change was weaker when we account for security values. Universalism was also negatively associated with relationship change (β = -.01, t = -2.19, p = .03, 95%CI (-.02, -.001)) with no interaction effect between anxiety and universalism. This shows a weaker association between anxiety and relationship change when we also include universalism values.

Regression analyses for friend and neighbour relationship change showed anxiety and security were negatively associated with relationship changes (β = -.04, t = -2.38, p = .02, 95%CI (-.08, -.01), β = -.01, t = -2.05, p = .04, 95%CI (-.02, -.0004), respectively), with the interaction effect between anxiety and security on relationship change also significant (β = .03, t = 3.21, p = .001, 95%CI (.13, .05). In order to further explore this interaction, we estimated moderation effects when security values are at ± 1 SD. Significant moderation effects were found only among those with -1SD (i.e., lower security) and average levels of security (Low: Effect = -.10, t = -3.48, p = .001, 95%CI (-.16, -.05), average: Effect = -.04, t = -2.38, p = .02, 95%CI (-.08, -.01)). No significant moderating effect was found among people with high security value (Effect = .02, t = .79, p = .43, 95%CI (-.03, .06)). Thus, the association between anxiety and relations with those from the outer circles of relationships was weakened by including security as a moderator, although this change was relevant at only average or lower levels of security. There was also an interaction effect for the association of anxiety and universalism with relationship change (β = .03, t = 3.27, p = .001, 95%CI (.01, .05). We further explored this applying equation estimating moderation effects when universalistic values are at ± 1 SD from the mean. At a universal value of -1 SD (i.e., lower universal), significant moderation was found (Effect = -.10, t = -3.39, p = .001, 95%CI (-.16, -.04)). No moderating effect was found for the universal value at 0 SD (Effect = -.03, t = -1.87, p = .06, 95%CI (-.07, .00)) and for +1 SD (Effect = .04, t = 1.47, p = .14, 95%CI (-.01, .08)). Hence the relationship between anxiety and relationship change was weakened by including universalism as a moderator, but this change was only relevant at lower levels of universalism.

## Discussion

Major stressful events, such as the COVID-19 pandemic, have the potential to amplify our relationships with others, both positively and negatively. Although, at the time of writing, the health impact of COVID-19 on Thailand has been relatively modest, lockdowns and curfews in this country threatened to significantly disrupt everyday life. In addition, increased unemployment [34] poses a significant threat to interpersonal relations [35, 36], while a recent history of political polarization threatened to compromise any national response to the pandemic [37]. Nevertheless, our national on-street survey indicated an overall enhancement of relationship partnerships and family relationships. Consistent with other studies noted [13, 21], the current study suggests that COVID-19 does not necessarily lead to the decline of relationships amongst romantic partnerships and families. Despite the challenges reported elsewhere for those balancing family and work [4], interpersonal enhancement was greatest amongst those married with children. These relationship changes were further associated with the personal values of security and universalism.

Research worldwide suggests that the COVID-19 pandemic has been associated with increased levels of anxiety and psychological distress [38–40]. In our Introduction we suggested that daily stressors during a national crisis can be disruptive to relationships with being in a close relationship no longer having the protective effect on mental health reported prior to this coronavirus [36, 39]. Drawing on the Social Convoy model to identify spheres of relations, we note that change was evident, and positive, in relations with romantic partners and family. However, we found only small effects for the associations between anxiety about infection and perceived control of the virus and relationship change, potentially questioning the anxiety-buffering function of the more inner circle of relationships during COVID-19 [11]. Personal values had a greater association with relationship change: with regard to the two personal values of Schwartz’s circumplex model used in our study, we found that relationship decline was greatest amongst those who highly valued security. Those who priorities security may be the most adherent to government guidelines that promote avoidance of contact with others during coronavirus [41]. In contrast, universalistic values were positively associated with relationship change outside of the couple, in line with the self-transcendent concern with the general welfare of others expressed by those who prioritise this value [24]. We note that these two values, at low/moderate levels, also act as buffers for outer circle relationships in associations between anxiety and relationship decline. Taken together, these results suggest the importance of personal values in the adaptation of individuals to a novel stressor.

Although age or sex generally had minimal impact on relationship change, married individuals with children benefitted from an improvement in their closest relationships compared to those who were single and reported enhanced interactions with their friends and communities compared to those without children. This may result from the high levels of collectivism in this relatively traditional society [31] which, although undergoing many changes, still places high value on the household and childcare [42, 43]. Given that opportunities for close interactions and allied behaviours may change rapidly as a threat changes [44, 45] such disparities could be profitably further explored in longitudinal work.

### Limitations

We recognise several limitations to our study. While our on-street survey had a good response rate (>80%), gathering data face-to-face in the midst of a pandemic meant we were restricted in the time we had with each respondent, and therefore the number of questions asked. Those who were sufficiently confident to interact with the survey researchers on the street may have had lower levels of anxiety about the virus. Further, we could not reach individuals and/or families who remained isolated within their homes and may be more vulnerable to both the virus and the relationship strains associated with being homebound [46]. Our data were self-reported, and we were not able to establish baseline relationship scores, or measure the buffering impact of interpersonal factors such as partner responsiveness [36]. Our data was cross-sectional, limiting our ability to study trends as the pandemic evolved, and the effects reported for overall relationship change were modest. More detailed information on the participants housing arrangements, and the format and regulation of social interactions over time, may have enhanced the significance of our findings.

### Implications and conclusions

The continuing challenges posed by COVID-19 are likely to have far-reaching impacts worldwide. For countries such as Thailand, whose economy is significantly reliant on tourism, economic uncertainties associated with the coronavirus are likely to persist for some time. Our findings however suggest that there is no clear spill-over of these stressors into interpersonal relations. The vast majority of Thai participants in this study reported that their relationships with others did not change because of the pandemic. This may partly result from the activity of grassroots organisations that played a central role in the Thai response, with a million village health volunteers across Thailand visiting homes to check temperatures and share health information [37], supported by allied community activities such as a new national food donation scheme [47, 48]. We note, however, that for those who prioritise security, or value self-promotion rather than universalistic values, there was evidence of less positive changes in relationships. Clinical practitioners might benefit from discussing these personal values with clients who find relationship processes difficult during this period. Public health teams may find it beneficial to address these values, potentially through communications that reassures individuals about the security of their society during this period. Such messages can be communicated via platforms most relevant to their audiences, employing appropriate influencers as appropriate [39].

We recognise that some of our findings may reflect the initial success of Thailand in tackling this pandemic. Research conducted online across China using similar questions found a sharper amplification of intimate relationship but greater decline in more distant interactions. In this Chinese sample relationship improvement was most evident for respondents in quarantine, and for those who were the most psychologically distressed [49]. These findings may reflect the impact of the more wide-ranging restrictions introduced in that country in the early phases of the pandemic, and the subsequent greater dependency amongst individuals and between communities.

Our findings reinforce the need to protect interpersonal relationship to help build resilience during this difficult time. In particular, personal relationships may be best protected by reassuring the wider population that personal and societal security can be defended through appropriate preventative actions. Whilst we did not directly address domestic violence in our study, we note that a lack of control over the pandemic was associated with a decline in partner relations, therefore also reinforcing the need for governmental authorities to provide a sense of reassurance over the personal actions individuals can take to remain safer during the pandemic. The training of local volunteers by Thai health authorities to enhance community resilience has been extended to provide vulnerable populations (e.g. those with special needs [50]); we would recommend any such scheme also takes note of the role of promoting universalistic values in enhancing positive relationships with individuals and communities beyond the closet social circles. Further research across cultures could profitably examine predictors of relationship functioning across cultures experiencing different levels of pandemic stress in order to better promote psychological well- being and daily functioning during this challenging time.

## Supporting information

S1 File The interview guideline and questionnaire items. (DOCX)
