# Supplementary material for: For better or worse: Relationship change in Thailand during COVID-19
Source: PLoS One. 2022 May 25;17(5):e0264614. doi: 10.1371/journal.pone.0264614 (PMC9132297; doi:10.1371/journal.pone.0264614)
Supplement: S1 File — (DOCX) [file pone.0264614.s001.docx]

**The interview guideline and questionnaire items**

Instruction to the interviewer

1. Researchers (fieldworkers) are trained and instructed to approach every 1 in 3 pedestrians passing a pre-determined point on regional shopping streets or near regional stations (rather than major shopping centers or major transportation stations as these already imply pedestrians’ willingness to mix with large crowds).
2. Researchers are assigned into different time slots so that some question respondents in the ‘quiet times’ e.g. the middle of the morning in a shopping street and some in the ‘busier time’ e.g. rush hours after work. We will note the time of day, date and location.
3. Introduce to respondents that you are part of University running a questionnaire related to the new virus that has been reported in Thailand. Provide respondents information as following: the interview takes no more than 5 minutes, the answers are confidential, name or other identifiers are not recorded. Respondents can stop answering the questions as soon as they like (as shown in the inform consent sheet).
4. Note if the person is willing to participate or not, as we need an exact response rate.
5. Researchers can work in pairs. However, each should try to collect at least 20 respondents who can answer all the questionnaire items.

Inform consent

We are inviting you to participate in a research study about your lifestyle change during COVID-19. Your participation to our study is completely voluntary. We are asking you because you are an adult aged 18 years old or older, not the vulnerable people; such as, pregnancy, or having any symptoms of flu, having Thai nationality, and willing to participate in our study. As we review the following information, please ask questions as they come to mind. If you have questions come to mind, please ask questions or contact the researcher(s) using the information that is enclosed below.

Research overview: You will be asked about your opinion or feeling about daily life during COVID-19 pandemic, your relationship with others and your demographic information, i.e., age, gender, marital status, and occupations. Completion of the survey takes only 3-5 minutes.

The purpose of this research is to better understand how individual factors, including personal values and perceived control over the virus could affect changes in relationships with surrounding people. Findings from this study will be used to generate presentations at professional conferences and to develop a manuscript for publication in a scientific journal.

To minimal risk to participants in the study, Interviewers used appropriate personal protective equipment (including facemasks and hand sanitizers) and maintained physical distance from interviewees, in line with guidance from Thai national health authorities.

This study has no compensation to participants, your answer will be recorded in the interviewer’s tablets via an online data collection system and is entirely anonymous. No identifiable information will be included. Data will be securely stored a private office space.

By agreeing to participate, you acknowledge that your participation in the study is voluntary, you are 18 years of age, and that you are aware that you may choose to terminate your participation in the study at any time and for any reason without losing any benefits.

----------------

o I consent, begin the study (1)

o I do not consent, I do not wish to participate (2)

----------------

Questionnaire items

Research name

Date:

Time of day:

City of the study (e.g. Bangkok) :

1. Demographics

- Sex (male/ female)
- Age in years
- Occupation (Blue collar, Student, Company worker, Retired, Unemployed, Freelance, Business owner, Housewife, Government officer, Others (specify)……………….)
- Marital status (Single, Married: no children, Married with children, Divorced/widowed: no children, Divorced/Widowed with children

1. How anxious are you about catching covid-19?
   1. Very anxious
   2. Moderately anxious
   3. Not at all anxious
2. How anxious are your closest friends and family members about contracting covid-19?
   1. Very anxious
   2. Moderately anxious
   3. Not at all anxious
3. How worried are you about accidentally infecting others with the virus (when you pass it on and maybe don’t realize you are ill)?
   1. Very anxious
   2. Moderately anxious
   3. Not at all anxious
4. How much control do you think over the likelihood of you getting infected by the virus?
   1. No control
   2. A little control
   3. A great deal of control
5. How has your relationship with other people changed during this time? Is your relationship a) better, b) the same or c) worse?
   - 1. With your romantic partner (if you don’t have one then say missing)
     2. With your family
     3. With your friends
     4. With others in your neighborhood
6. Finally, here are a few questions about yourself. How important are the following for you on a scale of 1-10 where 10 is high? How important for you is to:
7. Understand different people
8. For the government to make sure everyone is safe
9. To make sure people are treated equally and have equal opportunities
10. To live in safe and secure surroundings

At the end: Thank respondent for their time and explain that the questions are based on previous research showing that people often change their behaviors following a disease outbreak, sometimes in ways which are more helpful than others. The WHO recommends people to keep at least 1-2 meters distance from others and to regularly wash your hands, and avoid close contact, when possible, with anyone showing covid-19 symptoms (such as coughing and sneezing).
